# Cross-Protection against Lethal H5N1 Challenge in Ferrets with an Adjuvanted Pandemic Influenza Vaccine

**DOI:** 10.1371/journal.pone.0001401

**Published:** 2008-01-02

**Authors:** Benoît Baras, Koert J. Stittelaar, James H. Simon, Robert J. M. M. Thoolen, Sally P. Mossman, Frank H. M. Pistoor, Geert van Amerongen, Martine A. Wettendorff, Emmanuel Hanon, Albert D. M. E. Osterhaus

**Affiliations:** 1 Preclinical Virology, GlaxoSmithKline Biologicals, Rixensart, Belgium; 2 ViroClinics BV, Rotterdam, The Netherlands; 3 Global Pathology Support, Toxicologic Pathology, The Hague, The Netherlands; 4 Department of Virology, Erasmus Medical Center (MC), Rotterdam, The Netherlands; Institut Pasteur Korea, Republic of Korea

## Abstract

**Background:**

Unprecedented spread between birds and mammals of highly pathogenic avian influenza viruses (HPAI) of the H5N1 subtype has resulted in hundreds of human infections with a high fatality rate. This has highlighted the urgent need for the development of H5N1 vaccines that can be produced rapidly and in sufficient quantities. Potential pandemic inactivated vaccines will ideally induce substantial intra-subtypic cross-protection in humans to warrant the option of use, either prior to or just after the start of a pandemic outbreak. In the present study, we evaluated a split H5N1 A/H5N1/Vietnam/1194/04, clade 1 candidate vaccine, adjuvanted with a proprietary oil-in- water emulsion based Adjuvant System proven to be well-tolerated and highly immunogenic in the human (Leroux-Roels et al. (2007) The Lancet 370:580–589), for its ability to induce intra-subtypic cross-protection against clade 2 H5N1/A/Indonesia/5/05 challenge in ferrets.

**Methodology and Principal Findings:**

All ferrets in control groups receiving non-adjuvanted vaccine or adjuvant alone failed to develop specific or cross-reactive neutralizing antibodies and all died or had to be euthanized within four days of virus challenge. Two doses of adjuvanted split H5N1 vaccine containing ≥1.7 µg HA induced neutralizing antibodies in the majority of ferrets to both clade 1 (17/23 (74%) responders) and clade 2 viruses (14/23 (61%) responders), and 96% (22/23) of vaccinees survived the lethal challenge. Furthermore lung virus loads and viral shedding in the upper respiratory tract were reduced in vaccinated animals relative to controls suggesting that vaccination might also confer a reduced risk of viral transmission.

**Conclusion:**

These protection data in a stringent challenge model in association with an excellent clinical profile highlight the potential of this adjuvanted H5N1 candidate vaccine as an effective tool in pandemic preparedness.

## Introduction

Influenza pandemics occurring over the past centuries have cost the lives of many millions of people. The unprecedented spread of the highly pathogenic avian influenza virus (HPAI) of the H5N1 subtype among birds and mammals in the past decade and hundreds of reported zoonotic transmissions with a high case fatality rate, emphasised the need for worldwide pandemic preparedness [1]–[3]. The timely availability of a safe and effective pandemic vaccine will play a crucial role in efforts to combat this pandemic threat [4]–[6].

Mathematical modelling has demonstrated that the use of a pre-pandemic vaccine before or soon after the onset of a pandemic, in combination with other protective interventions, can be highly effective in reducing the clinical attack rate by as much as 75% [7], [8]. Pre-pandemic vaccination strategies are supported by the results obtained during the re-appearance of H1N1 in 1976/77 which afforded the opportunity for vaccine trials in naïve and primed human subjects. In these studies, the outcome was an improved responsiveness in primed individuals compared to naïve individuals upon vaccination with the then newly-emerged H1N1 strain [9]. Extensive genetic characterization of HPAI H5N1 strains has elucidated the natural evolutionary relationship of these strains, linking groups known as ‘clades’ to a common ancestor [10]. Reciprocal cross-reactivities in heamagglutination inhibition (HI) tests have demonstrated antigenic similarities of heamagglutinin molecules (HAs) within the same genetic clade and have distinguished representatives of different clades [10]. The efficacy of a pre-pandemic inactivated vaccine relies on its ability to induce an immune response that will protect against a future pandemic influenza virus strain. Since it is not possible to predict the nature of the pandemic virus strain, the feasibility of a pre-pandemic vaccination strategy will largely depend on the breadth of the immune response and protection that is induced following administration of such a vaccine. The production of such a candidate inactivated pre-pandemic vaccine using a viral strain derived from a currently circulating avian H5N1 strain is being considered an attractive strategy [11], [12].

Recently, Leroux-Roels and colleagues [13] investigated the safety and immunogenicity of an inactivated split A/Vietnam/1194/2005 (clade 1) H5N1 pandemic candidate vaccine adjuvanted with a proprietary oil-in-water emulsion based Adjuvant System in healthy human adults aged 18–60 years. This study was the first to show robust immune responses induced at low antigen doses in association with a novel adjuvant, including the induction of cross-clade immunity against a drifted H5N1 isolate (A/Indonesia/5/2005, clade 2) [4], [13]. This adjuvanted H5N1 candidate vaccine was well-tolerated by all trial participants [13].

As cross-protective efficacy studies of an H5N1 candidate vaccine cannot currently be investigated in clinical trials, for obvious ethical reasons, an animal model was used to evaluate the vaccine. Here, the same inactivated split A/Vietnam/1194/2005 (clade 1) H5N1 adjuvanted vaccine was evaluated in the ferret (*Mustela putorius furo*) for its potential to induce efficient cross-protective immunity against a clade 2 drifted strain (A/Indonesia/5/2005). Although the mouse is the most suitable animal model for evaluation of influenza vaccine-induced antigen-specific T cell responses, the ferret is currently accepted as the most suitable mammalian host for efficacy studies of HPAI H5N1 vaccines [14], [15]. As recently shown by Govorkova *et al.* [16], the ferret model provides the basis for developing influenza vaccines that will be effective in the face of a contemporary influenza pandemic threat. To ensure that immunological cross-reactivity and cross-protection would be evaluated in sufficiently stringent conditions, the preclinical study reported here was performed using virus strains from different clades.

## Results and Discussion

Four groups of 6 ferrets were immunized intramuscularly with two doses of 15, 7.5, 3.8 or 1.7 µg HA of inactivated split A/H5N1/Vietnam/1194/04 NIBRG-14 (recombinant clade 1 H5N1 engineered by reverse genetics) vaccine adjuvanted with a proprietary oil-in-water emulsion based Adjuvant System [13]. The two control groups included ferrets administered with either the Adjuvant System alone or the non-adjuvanted A/Vietnam vaccine (containing 15 µg HA). Ferrets were vaccinated on days 0 and 21 and challenged intratracheally on day 49 with a lethal dose of A/Indonesia/5/2005 virus, 10^5^ TCID_50 _(50% tissue culture infective dose). Following the challenge with A/Indonesia all control animals receiving adjuvant alone or non-adjuvanted A/Vietnam vaccine died or were moribund and were euthanized on days 3 or 4 (Table 1). In contrast, all ferrets that received two doses of ≥3.8 µg of the adjuvanted A/Vietnam vaccine survived the lethal heterologous challenge. Furthermore, all except one animal survived the challenge in the group of ferrets who received the lowest dose (1.7 µg HA) of the adjuvanted vaccine. Thus overall 96% of animals immunized with adjuvanted H5N1 split vaccine were protected against the lethal challenge with A/Indonesia and survived to the end of the challenge phase on day 5 (see Table 1).

**Table 1:** Efficacy of adjuvanted split H5N1-vaccine against a heterologous H5N1 challenge in ferrets.

Vaccination regimen	Dead/Total (% survival^c^)	Viral load in the lung^a^		Viral shedding in the URT^b^	
		Ferrets (%) with viral load ≤10^2^	Ferrets (%) with viral load ≥10^5.5^	Ferrets (%) with viral shedding	Ferrets (%) with viral titer ≥10^2^
Adjuvant alone	6/6 (0)	0/6 (0)	6/6 (100)	6/6 (100)	5/6 (83)
Unadjuvanted H5N1 (15 µg)	6/6 (0)	0/6 (0)	6/6 (100)	5/6 (83)	5/6 (83)
Adjuvanted H5N1 (1.7 µg)	1/6 (83)	4/6 (67)	0/6 (0)	2/6 (33)	2/6 (33)
Adjuvanted H5N1 (3.8 µg)	0/6 (100)	3/6 (50)	0/6 (0)	1/6 (17)	0/6 (0)
Adjuvanted H5N1 (7.5 µg)	0/5 (100)	4/5 (80)	0/5 (0)	1/5 (20)	0/5 (0)
Adjuvanted H5N1 (15 µg)	0/6 (100)	5/6 (83)	0/6 (0)	2/6 (33)	2/6 (33)

a TCID_50_ per gram of lung tissue on Day 5 post-challenge or the day of the death

b Virus titration (TCID_50 _per ml of swab) in the Upper Respiratory Tract (URT) from throat and nasal swabs collected on days 2,3,4 and 5 post-challenge until day of death.

c % survival on day 5 post challenge

Moribund animals showed general depression, anorexia, lethargy and exhibited clinical signs of respiratory disease, including dyspnea. All animals that died prematurely showed signs of atypical pneumonia in one or more lung lobes by macroscopic and/or microscopic analysis (data not shown).

**Figure 1:**
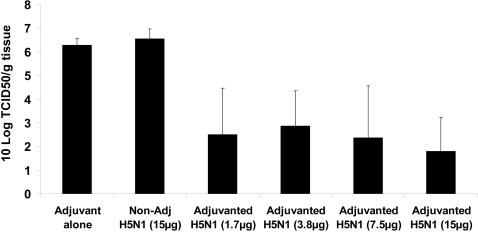
A/H5N1 viral load in the lung:

High levels of virus replication (≥10^5.5^ TCID_50_/g tissue) were observed in the lungs of all ferrets immunized with either the Adjuvant System alone or with non-adjuvanted A/Vietnam vaccine. Conversely, in 67% ferrets immunized with the adjuvanted A/Vietnam vaccines, lung virus loads were <10^2^ TCID_50_/g of lung tissue (Table 1). These low virus loads were observed in 80% and 83% of ferrets immunized with the adjuvanted 7.5 µg and 15 µg formulations, respectively. However, there was no overall antigen-dose dependent effect on viral load observed among ferrets immunized with adjuvanted A/Vietnam vaccines (Figure 1). In general high levels of virus replication in the lung correlated with mortality. Of the animals that died all had a lung virus load ≥8×10^4^ TCID_50_/g tissue, and there was only one animal with a high viral load (1×10^5^ TCID_50_/g tissue) who survived until the end of the experiment (day 5 post-challenge).

Both the amount of virus shed into the upper respiratory tract and numbers of animals shedding virus were reduced in vaccinees relative to control animals. A majority (92%) of ferrets inoculated with the Adjuvant System alone or the non-adjuvanted A/Vietnam vaccine shed high levels of virus (>10^2^ TCID_50_ /ml) in the upper respiratory tract (throat or nasal swabs) throughout the course of infection. Conversely, only 26% ferrets receiving adjuvanted A/Vietnam vaccines shed virus in throat or nasal swabs and none of the ferrets immunized with the 3.8 or 7.5 µg doses of adjuvanted A/Vietnam vaccines exhibited viral shedding >10^2^ TCID_50 _/ml (Table 1). Since the probability of viral transmission would likely decrease with reduced virus shedding in the upper respiratory tract [17], [18], our data suggest a potential for vaccination to confer a lower risk of viral transmission, a key property in controlling pandemic virus spread within populations.

Before vaccination, ferrets used in this study were influenza naïve as measured by an ELISA assay for the presence of antibodies specific for nucleoprotein [19]. Post-vaccination serological assessments showed that the adjuvanted A/Vietnam vaccine formulations induced neutralizing antibody responses against the homologous A/Vietnam strain with 74% of responders (ferrets with neutralizing antibody titres > 28) compared to 100% non responders in control groups (Table 2). Furthermore, the adjuvanted A/Vietnam vaccine induced inter-clade cross-neutralizing antibody responses to the heterologous A/Indonesia clade 2 strain (Table 2) with 61% responders, while no neutralizing antibody response (<28) was observed in ferrets immunized with the non-adjuvanted A/Vietnam vaccine or the Adjuvant System alone. No antigen-dose dependent effect on neutralizing antibody titres was observed amongst ferrets immunized with adjuvanted A/Vietnam vaccines. Interestingly, all animals without a detectable neutralizing antibody response to A/Vietnam or to A/Indonesia exhibited lung virus load > 8×10^3^ TCID_50_/g tissue, whereas 94% (16/17) of ferrets with anti-A/Vietnam neutralizing antibody response and 93% (13/14) with anti-A/Indonesia responses showed virus loads in their lungs below 10^2^ TCID_50_/g tissue. Of 22 animals surviving until the termination of the experiment at day five post-challenge, 17 (77%) were positive for the induction of an H5N1 neutralizing antibody response. Four of the five non-responder animals that were protected from mortality exhibited reduced virus loads in the lung relative to unvaccinated control animals (ranging from 9×10^3^ to 5×10^4^ TCID_50_/g tissue), suggesting a possible role for vaccine induced cellular immune responses in the control of virus replication.

**Table 2:** Neutralizing antibody responses to the vaccine strain (A/Vietnam) and the challenge strain (A/Indonesia) 42 days after first vaccination, i.e 21 days after second vaccination

Vaccination regimen	Anti-A/Vietnam neutralizing titers		Anti-A/Indonesia neutralizing titers	
	Day 21(Post II)^a^	Responders^b^	Day 21 (Post II)^a^	Responders^b^
	GMT (95% CI)		GMT (95% CI)	
Adjuvant alone	<28	0/6	<28	0/6
Unadjuvanted H5N1 (15 µg)	<28	0/6	<28	0/6
Adjuvanted H5N1 (1.7 µg)	83 (19–371)	4/6	36 (15–83)	4/6
Adjuvanted H5N1 (3.8 µg)	113 (24–521)	4/6	43 (16–116)	4/6
Adjuvanted H5N1 (7.5 µg)^a^	104 (18–602)	4/5	35 (12–107)	3/5
Adjuvanted H5N1 (15 µg)	83 (29–234)	5/6	26 (12–55)	3/6

a Neutralizing antibody GMT were <28 before immunization (Day 0) and after first vaccination (Day 21 Post I).

b A responder is defined by neutralizing titers ≥ 28.

These results highlight the potential of this adjuvanted split H5N1 candidate vaccine to induce, even with a low dose of antigen (3.8 µg), a strong cross-protective response in ferrets against a lethal challenge with heterologous H5N1 virus from another genetic sublineage and suggest that cross-protection may be mediated at least in part by antigen-induced humoral immunity. However, we cannot rule out a role for cell mediated immune responses in cross-protection in the ferret model. There is evidence that cell-mediated immune responses can be linked to protection against influenza in humans [20], [21] and it has been shown in other disease systems that adjuvants can be effective at inducing protective cell mediated immune responses [22]–[25]. As immunological reagents needed to study T cell responses in ferrets are largely lacking, investigation of influenza vaccine-induced antigen-specific T cell responses will be undertaken in mouse and macaque models and in clinical studies.

Data reported in the literature and other preliminary investigations (unpublished observations) suggest that multiple mechanisms of action can account for the immunostimulatory properties of the adjuvant used in this study. Indeed, in addition to their vehicle properties, oil-in-water based emulsions have been shown to induce local inflammation and to attract immunocompetent cells to the injection site [26], [27]. In this context, studies are currently ongoing to elucidate the adjuvanted vaccine's ability to induce cell mediated immune responses.

Other published studies have similarly documented cross-clade protection in ferrets vaccinated with different pandemic vaccine candidates [16], [28], [29]. Here we document the first study in which protection against heterologous challenge in ferrets is generated by a candidate pandemic vaccine proven to be safe and immunogenic in humans [13], inducing neutralizing antibodies specific for both the vaccine strain and cross-reactive to the heterologous H5N1 virus from a distant clade. These parallel sets of encouraging data with the same product suggest the development of a safe and effective pre-pandemic vaccine is a realistic goal.

## Materials and Methods

The study was carried out with outbred adult female ferrets (Mustela putorius furo: age approximately 8 months, bodyweight 0.8–1.5 kg; Schimmel, Uddel, The Netherlands) in accordance with the institutional guidelines for care and use of laboratory animals. The AS adjuvanted candidate vaccine used in this study was A/H5N1, inactivated, split influenza vaccine formulated with a proprietary oil-in-water emulsion based Adjuvant System manufactured by GlaxoSmithKline (GSK) Biologicals, Branch of SmithKline Beecham Pharma GmbH & Co. KG (Dresden, Germany) [13]. The four experimental groups of ferrets corresponded to immunization with vaccine containing four dose levels of inactivated split A/Vietnam HA (1.7, 3.8, 7.5 or 15 µg HA). Two control groups included ferrets administered with either the Adjuvant System alone or the highest dose (15 µg HA) of non-adjuvanted A/Vietnam vaccine and were. Ferrets were vaccinated on days 0 and 21 and were then challenged by the intra tracheal route on day 49 with a lethal dose (105 TCID50 or 50% Tissue Culture Infective Dose) of A/Indonesia/5/05 (H5N1 clade 2). All surviving animals were euthanized on day 54. One animal in the experimental group vaccinated with 7.5 µg of adjuvanted vaccine that was not challenged in compliance with protocol guidelines was excluded from the data recording process. Viral titrations were performed as described elsewhere [30]. Briefly, pharyngeal and nasal swabs were collected from all animals at days 1, 2, 3, 4, 5 post-challenge; after necropsy cranioventral, craniodorsal, caudoventral and caudodorsal sections of the right lung from each animal were collected and weighed. Lung sections and individual swabs were homogenized and resuspended in 3 ml medium and stored at −80°C until analysis. Viral titres were determined by means of virus titration culture on Madin-Darby canine kidney (MDCK) cells. Data were expressed as log TCID50 per gram of lung tissue or per ml of swabs.

Neutralizing antibodies were determined in a microneutralization assay on thawed frozen serum samples as described previously [13]. A serologic response corresponds to a neutralizing titre >28.
